# Correlation between Immunohistochemical and Histomorphological Features of Ampullary Carcinomas: A Study on 72 Cases from a Tertiary Health Care Center

**DOI:** 10.1155/2020/2080351

**Published:** 2020-01-06

**Authors:** Usha Mary Abraham, Subramaniam Ramkumar

**Affiliations:** ^1^Department of Pathology, Amala Institute of Medical Sciences, Amala Nagar PO, Thrissur, 680555 Kerala, India; ^2^Department of Pathology, Woodland Hospital, Dhankheti, East Khasi Hills, Shillong, Meghalaya 793003, India

## Abstract

Tumors involving the ampulla could be arising primarily in the ampulla or extending from the adjacent. When a neoplasm is centered primarily in the ampulla with or without periampullary mucosal involvement, it is considered a primary ampullary carcinoma. These tumors generally have a better prognosis than duodenal and pancreaticobiliary neoplasms secondarily involving the ampulla. Distinguishing between the two primary types has prognostic implications, as the pancreaticobiliary-type ampullary carcinomas are said to have a poorer prognosis than the intestinal-type. Morphological analysis alone may not suffice in this context. The role of immunohistochemistry has therefore been explored by various groups of workers. The purpose of the present study was to determine the role of morphology and cytokeratin profile in accurate typing of ampullary carcinomas as intestinal and pancreaticobiliary.

## 1. Introduction

Junctions of two different types of epithelia can give rise to tumors that may show features of either the involved epithelia or a mixture or an intermediate amount of both. One such junction is the ampulla of Vater [1, 2]. Primary ampullary carcinomas generally have a better prognosis than duodenal and pancreaticobiliary neoplasms that secondarily involve the ampulla [3, 4]. On the basis of microscopic appearance, primary ampullary carcinomas are classified as intestinal, pancreaticobiliary, or mixed types [3, 4]. Distinguishing between the first two types is important: The pancreatobiliary-type carries a poorer prognosis than the intestinal-type [5, 6]. Morphological analysis alone may not be sufficient for diagnosis. The role of immunohistochemistry has therefore been explored by various groups of workers. The pattern of cytokeratin (CK) expression, CK7−/CK20+ in the intestinal-type and CK7+/CK20– in the pancreaticobiliary-type, correlated with morphological characteristics in some of these studies [7, 8]. However, results of other studies contradicted these observations [9, 10]. The purpose of this study was to determine the role of cell structure and CK profile in accurate typing of ampullary carcinomas as intestinal and pancreaticobiliary.

## 2. Materials and Method

Seventy-two specimens of ampullary carcinoma were studied in the Department of Pathology, in a tertiary health care center in India, over a period of 8 years. Of these, 25 were obtained through pancreaticoduodenectomy and 47 through endoscopic biopsy. After the relevant paraffin sections and blocks were screened, 30 cases were selected for this study, according to the following criteria:

1. Histopathological features were unequivocally those of a pure, invasive adenocarcinoma
2. The neoplasm involved primarily the ampulla of Vater, with or without periampullary extension, as judged by gross examination of the resection specimens and by the endoscopic appearance

Specimens with an exclusively periampullary pattern of growth were not included in the study. Clinical data included the mode of presentation and endoscopic or operative findings, obtained from case files and pathology request forms. Gross, microscopic, and immunohistochemical findings were recorded in the pathological examination.

### 2.1. Gross Examination

#### 2.1.1. Resection Specimens

For each specimen, we recorded the location and pattern of growth (ampullary or mixed ampullary/periampullary); size of the lesion; gross morphological type (i.e., ulcerative, polypoid, or stenosing); and involvement of the head of the pancreas, duodenal wall, common bile duct, and lymph nodes.

#### 2.1.2. Endoscopic Biopsy Samples

Endoscopic observation yielded information regarding the pattern of growth, size, and gross morphological type. This information was collected through pathology request forms or from patient files.

### 2.2. Microscopic Examination

We examined the architecture of the neoplasm (e.g., tubular, papillary, or mixed) cell structure (including cell height), the presence of nuclear atypia, and the nature of the stroma. We attempted to classify each of the tumors as intestinal or pancreaticobiliary or of mixed-type.

### 2.3. Immunochemistry

Immunostaining for CK7 and CK20 was performed on sections from all 30 blocks by means of the following technique [11]. First, two specimens of colonic carcinoma and one of cholangiocarcinoma were chosen as controls for CK7 and CK20, respectively. From the chosen paraffin blocks, 4 *μ*m thick sections were made and then stained with hematoxylin and eosin and visualized by microscopy. To detect CK7 and CK20 expression, we performed immunohistochemical studies with horseradish peroxidase (HRP), a supersensitive polymer, along with appropriate controls. We used 3,3′-diaminobenzidine tetrahydrochloride as the chromogen. By using a pressure cooker for 10 min and citrate buffer at a pH of 6.0, we retrieved the antigens to CK7 and CK20 (BioGenex, San Ramon, CA). The sections were then assessed microscopically. Scoring of nuclear immunoreactivity was performed in the well-stained area with no interference by nonspecific staining background. Immunoreactivity was graded according to the percentage of cells expressing the marker: 0 (no cells expressed the marker), 1 (<25% of neoplastic cells showed positivity), 2 (25%–50% of neoplastic cells showed positivity), and 3 (>50% of neoplastic cells showed positivity). The intensity of staining was also assessed, although in a subjective manner, and graded from 1+ to 3+ by two of the authors independently (UA and VN), and their results were compared.

## 3. Results

**Figure 1:**
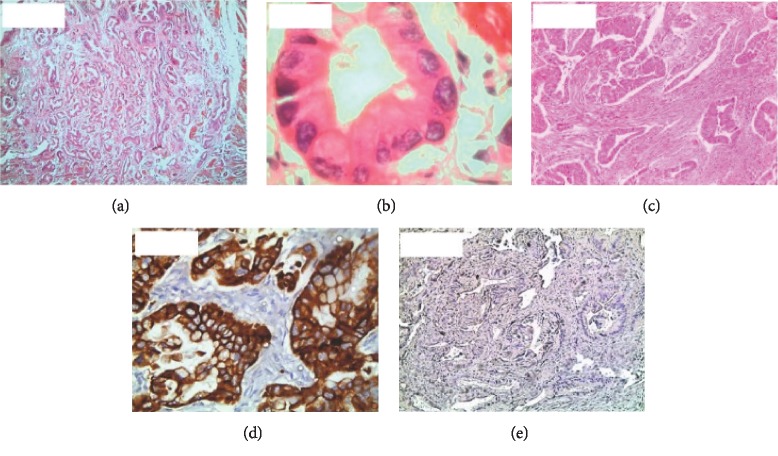
(a) Pancreatobiliary-type carcinoma with a tubular pattern (hematoxylin-eosin, 40x). (b) Pancreatobiliary-type carcinoma with marked nuclear atypia (hematoxylin-eosin, 400x). (c) Pancreatobiliary-type carcinoma with desmoplasia (hematoxylin-eosin, 100x). (d) Pancreatobiliary-type carcinoma with CK7 positivity (immunohistochemistry, 400x). (e) Pancreatobiliary-type carcinoma with CK20 negativity (immunohistochemistry, 100x).

**Figure 2:**
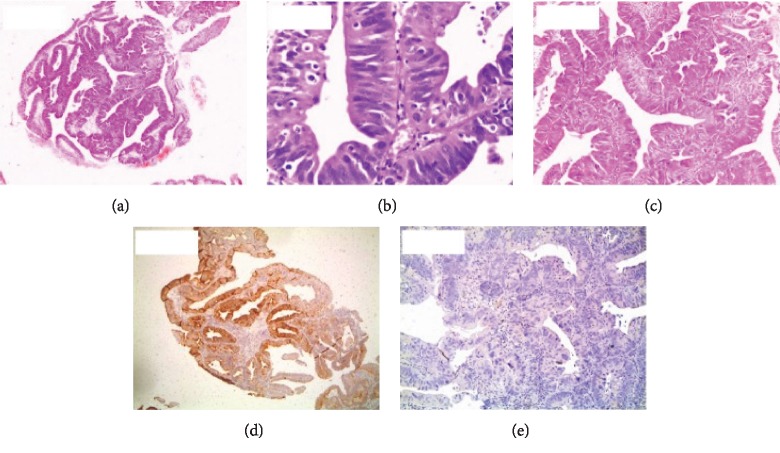
(a) Intestinal-type carcinoma with a tubulopapillary pattern (hematoxylin-eosin, 100x). (b) Intestinal-type carcinoma with minimal nuclear atypia (hematoxylin-eosin, 400x). (c) Intestinal-type carcinoma with the absence of desmoplasia (hematoxylin-eosin, 400x). (d) Intestinal-type carcinoma with CK20 positivity (immunohistochemistry, 100x). (e) Intestinal-type carcinoma with CK7 negativity (immunohistochemistry, 100x).

**Figure 3:**
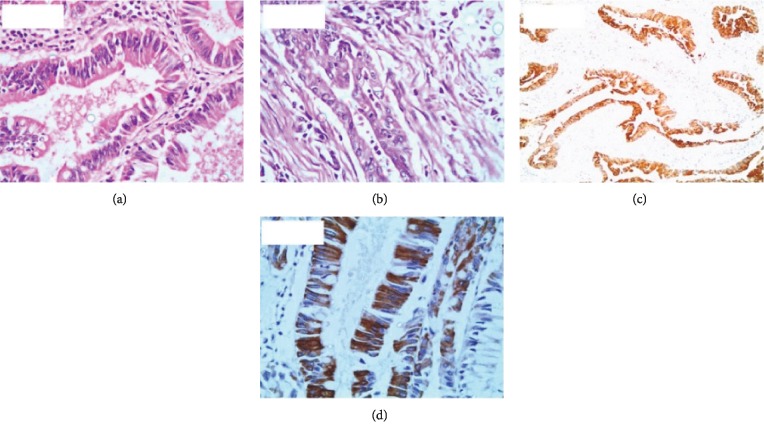
(a) Tall columnar cells in mixed-type carcinoma (hematoxylin-eosin, 400x). (b) Cuboidal cells in mixed-type carcinoma (hematoxylin-eosin, 400x). (c) CK7 positivity in mixed-type carcinoma (immunohistochemistry, 100x). (d) Ck20 positivity in mixed-type carcinoma (immunohistochemistry, 400x).

Of the specimens studied, 21 showed correlations between morphological type and CK7/CK20 staining pattern. Of these, two were of the intestinal-type of ampullary carcinoma (Figure 1(a)–1(e)), 14 were of the pancreaticobiliary-type (Figure 2(a)–2(e)), and five showed mixed intestinal-pancreaticobiliary morphological type and CK profile (Tables 1 and 2; Figure 3(a)–3(d)). In the remaining nine cases, the CK profile did not match cell structure (Tables 3 and 2).

The assessment of CK7 and CK20 expression revealed that of the nine metastatic tumors, 100% were CK7+, but only 44% (four) were CK20+. Among the six tumors without metastasis in our series, five (83%) expressed CK20, and four (67%) expressed CK7. This is in agreement with the earlier observations that colorectal adenocarcinomas with lymph node metastasis showed more CK7 expression than tumors without metastasis [4, 6].

### 3.1. Lymph Node Metastasis and Tumor Types

A total of 15 specimens obtained from Whipple's resections were included in this study, of which nine showed metastatic deposits in the lymph nodes. Of the nine specimens, only seven had a morphological type that was correlated with CK profile, which enabled subtyping of the tumors. Of the seven carcinomas with lymph node metastasis, four were of pancreaticobiliary-type and three were of mixed-type. Pure intestinal carcinomas did not show lymph node involvement. Of the two cases with no morphological-immunohistochemical correlation, which had also metastasized to lymph nodes, one had a pancreaticobiliary CK profile and an intestinal morphological appearance, and the other had a mixed CK profile and a pancreaticobiliary morphological appearance. Regardless of morphological subtypes, we observed that of the nine tumors with lymph node metastasis, all expressed CK7, whereas only four (44%) expressed CK20. Of the six tumors without metastasis, five (83%) expressed CK20, and four (67%) expressed CK7.

In this study, we attempted to find correlations of individual histological features (such as architecture, predominant cell type, nuclear grade, and degree of desmoplasia) with CK7 and CK20 expressions. We found that tumors with an exclusively tubular architecture always expressed CK7, but not CK20.

With regard to cell height, CK20 expression was correlated strongly with tall columnar cells; all CK20+ tumors had a tall columnar cell component.

### 3.2. Cell Height and Cytokeratin Expression

Twelve of the neoplasms assessed consisted of cuboidal or low columnar cells or both types. Eleven of these had a CK7+/CK20− profile, and the other had a CK7−/CK20− profile. Seven of these had a CK7+/CK20− profile, eight had a CK20+/CK7+ profile, and three had a CK7−/CK20+ profile. Although CK7 expression showed no significant correlation with cell height, all 11 CK20+ tumors had a considerable proportion of tall columnar cells.

The distribution of the different nuclear grades did not differ between CK7+ and CK20+ tumors. However, 100% of high nuclear grade (+++) neoplasms expressed CK7, and 20% expressed CK20. This suggests a correlation between CK7 expression and high grade; however, the majority of tumors in this study were either pancreatobiliary or of mixed-type and were CK7+. Observations about desmoplasia also followed a similar pattern. The results should be viewed with regard to the fact that majority of the tumors were CK7+.

### 3.3. Nuclear Grade and Cytokeratin Profile

All of the ten tumors with +++ nuclear grade showed CK7 expression. Of the 11 tumors with CK20 expression, nine had a low to medium nuclear grade (Tables 4 and 2). Among the 12 tumors with grade 2 to 3+ desmoplasia, CK7 expression was present in 11 (92%), and CK20 was present in 5 (42%). In tumors with 0–1+ desmoplasia, 83% expressed CK7 (15/18) and 33%, CK20 (6/18; Tables 5 and 2).

Our findings are important because accurate histological typing of ampullary carcinomas would provide information that guides staging and planning of operative procedures. It would also provide a solid foundation for analyzing pathogenetic mechanisms of these tumors by more advanced methods, such as molecular studies.

## 4. Discussion

Ampullary carcinomas constitute 5% of gastrointestinal carcinomas and usually occur in people older than 60 years. The clinical symptoms and signs include jaundice, itching, loss of appetite, loss of weight, pale colored stools, and other signs of biliary obstruction [12]. Ampullary carcinomas, although usually small at the time of diagnosis, present with early obstructive symptoms thereby resulting in early detection.

Most of these tumors are present as a small mass projecting into the duodenal lumen or as periductal thickening or bulging of the papilla. Intra-ampullary tumors extending into the duodenal mucosa can present as an exophytic or ulcerative growth [13, 14]. The spread of these tumors may be invasive or noninvasive. Noninvasive spread includes intramucosal spread to the duodenum as well as the proximal areas of the common bile and pancreatic ducts. The invasive tumors spread via the musculature into the adjacent duodenal and/or pancreas. The lymph nodes involved are the peripancreatic group. Distant metastasis is usually to the liver, also to the peritoneum, lungs, and pleura. Vascular and perineural invasions are also known to occur in these tumors [15, 16].

Pathologic staging is difficult due to the complexity of the anatomy of the ampulla of Vater. Ampullary carcinomas have been staged according to the AJCC Cancer Staging Manual 6th edition [13]. Based on the primary tumor (T), there are seven stages. Pancreaticoduodenectomy is the treatment of choice and the mainstay of therapy [3, 4, 17].

They can arise in the ampullary duodenal aspect, lined by intestinal mucosa, or in the deeper aspect, lined by pancreatic or bile duct mucosa. Morphologically, the two types resemble their colonic and pancreatic counterparts [11]. The intestinal variant consists of elongated tubular units lined by cells ranging in height from tall columnar to cuboidal, with basal nuclei and apical mucin. The immunohistochemical marker profile of these tumors is similar to that of colonic carcinomas (CK20+, CK7−, and mucin 2 positivity (MUC2+)) [13]. The pancreatobiliary variant has simple or branching glands lined by epithelium ranging in height from cuboidal to low columnar. Its immune profile resembles that of pancreatic carcinomas (CK7+, CK20−, and MUC2−) [13]. The intestinal variant has a better prognosis than pancreatobiliary subtypes [18].

However, the ampulla has complex histomorphological features, and the transition from pancreatobiliary ductal epithelium to intestinal epithelium is very subtle. Morphological overlaps are common and pose challenges in tumor classification. Further staging (T1/T2 or T3) is difficult due to mucosal invasion by these tumors and the occurrence of rare pancreatic acinar in the walls of the ampulla. Hence, a multifaceted diagnostic correlational approach among tumor morphological type, immunohistochemistry (CK and mucin immunoprofiling), and imaging studies can help circumvent the pitfalls in diagnosing ampullary carcinomas. Several studies with this multifaceted approach have been conducted; their focus was on the histomorphological features in varying combinations.

Zhou et al. were the first to show agreement between the histological classification and immunohistochemical characterization based on CKs. They found a high specificity of CK7 for pancreatobiliary carcinomas and of CK20 for intestinal carcinomas [7]. Similar findings were confirmed by the studies of Le Pessot et al. [8] and Roh et al. [15]. Furthermore, the findings of Westgaard et al., who studied 114 resection specimens, indicated similar moderate agreement between morphological type and CK immunoprofiling. They found that histological typing significantly discriminates between the pancreatobiliary and intestinal types of pancreatic head adenocarcinomas, which is important for determining prognosis [18]. CK7 and CK20 expressions could also have some bearing on the prognosis of carcinomas, regardless of subtypes. At least in one series on colorectal carcinomas, CK20 was expressed more in low-grade tumors and CK7 was expressed more in high-grade tumors with lymph node metastasis [17].

This study was an attempt to find correlations between the morphological type and CK profile of ampullary adenocarcinomas. Both CK7 and CK20 are simple CKs with restricted tissue and neoplastic distribution. In this study, 70% of ampullary carcinomas showed ampullary adenocarcinomas are correlated to an extent with CK profile, and morphological-immunohistochemical subtyping is possible in 70% of cases. Lymph node metastasis occurs more frequently with CK7+ tumors than with CK20+ tumors. However, the above fact is hypothesized due to the limitation of the number of cases evaluated. This can be further evaluated by performing relevant studies. Among the microscopic features, an exclusively tubular architecture is associated with CK7 expression. Tall columnar cell component is unequivocally related to CK20 expression.

The correlations between some of the individual microscopic features, such as architecture and cell height with CK7 and CK20 expressions, are greater than those between intestinal and pancreatobiliary morphological types and combined CK7-CK20 profile.

## Figures and Tables

**Table 1:** Morphological and immunohistochemical correlations in the subtypes of ampullary carcinoma.

Morphological type	Cytokeratin profile	No. of cases
Intestinal	CK7−/CK20+	2
Pancreaticobiliary	CK7+/CK20−	14
Mixed	CK7+/CK20+	5
Total		21

**Table 2:** 

					Gross findings				Extent of spread		Microscopic findings					Immunohistochemistry					
Serial no.	Biopsy number	Age/sex	Clinical details	Radiological findings	Type of specimen	Pattern of growth	Size	Morphology	Local extent of spread	Lymph node involvement	Architecture	Cell height	Nuclear atypia	Stromal desmoplasia	Impression	Extent	Intensity	Extent	Intensity	Inference in IHC	Correlation
1	2414/10	48/F	Obstructive jaundice for 6 months	CT shows a small enhancing lesion in D2. Endoscopy shows bulky ampulla with small ulcer	Whipple's specimen	Intra-ampullary	1 × 1 cm	Nodular mass	Infiltration into pancreas seen	1/6	Tubule, trabeculae	Low columnar	--	--	Pancreatobiliary	3	++	--	--	Pancreatobiliary	Present

2	2041/10	58/F	Obstructive jaundice for 3 months	Endoscopy shows fleshy ampulla	Whipple's specimen	Intra-ampullary	0.8 × 0.5 cm	Nodular mass	Infiltration into wall of duodenum and bile duct	-	Tubule, papillae, solid pattern	Tall columnar	+	--	Pancreatobiliary	3	++	--	--	Pancreatobiliary	Present

3	2208/09	44/M	Jaundice-3 weeks	Endoscopy shows nodular mass	Whipple's specimen	Periampullary	3 × 1 cm	Nodular mass	Infiltration into wall of duodenum and bile duct	—	Tubules, papillae	Tall columnar	+ to +++	-	Intestinal	3	+++	--	--	Pancreatobiliary	x

4	1134/10	55/F	Obstructive jaundice	Endoscopy shows prominent ampulla	Endoscopic biopsy	Intra-ampullary	-	-		-	Tubules	Cuboidal	--	+	Pancreatobiliary	3	+++	-	--	Pancreatobiliary	Present

5	1937/10	59/F	Jaundice for 3 weeks	CT shows periampullary mass with dilated CBD	Whipple's specimen	Periampullary	4 × 3 cm	Ulceroproliferative growth	Infiltration into duodenal wall and focally in to pancreas	-	Tubules, papillae	Tall columnar	+	+	Intestinal	--	--	3	--	Intestinal	Present

6	1883/07	40/M	Jaundice 1 month	Endoscopy showed a friable prominent ampulla	Whipple's specimen	Intra-ampullary	2 × 3 cm	Friable nodular mass	Infiltration into pancreas and duodenal wall	1/4	Tubules, cords, and groups	Low columnar	++	+++	Pancreatobiliary	3	+++	--	--	Pancreatobiliary	Present

7	8707/10	72/male	Jaundice 2 weeks. Known case of tuberculosis	Endoscopy showed a periampullary growth	Endoscopic biopsy	Periampullary	-	-	-	-	Tubules, papillae, focal cribriform	Low columnar to tall columnar	+ to++	+	Pancreatobiliary	3	+++	--	--	Pancreatobiliary	Present

8	8764/10	57/male	Recurrent pancreatitis, abdominal pain 6 months	Endoscopy large ampullary growth	Endoscopic biopsy	Ampullary	1 cm	Nodular mass	—-	—-	Tubules, papillae	Tall columnar	++	-	Intestinal	--	--	2	+++	Intestinal	Present

9	S810/10	38/male	Jaundice 2 weeks	Endoscopy polypoid pedunculated mass	Whipple's specimen	Periampullary	5 × 3 cm	Polypoid pedunculated mass	—	2/8	Tubules, papillae	Low columnar to tall columnar	++	++	Intestinal/pancreatobiliary	3	+++	2	++	Intestinal/pancreatobiliary	Present

10	S1184/10	78/male	Obstructive jaundice, abdominal pain 1 month	USG-mass in the head of the pancreas, endoscopy-prominent ampulla	Whipple's specimen	Ampullary	1.9 × 1.5 cm	Ill circumscribed firm bulky mass	Infiltration in to duodenal wall	-	Tubules, cribriform and solid pattern	Cuboidal to tall columnar	+ to ++	++	Intestinal/pancreatobiliary	3	+++	3	++	Intestinal/pancreatobiliary	Present

11	S1444/10	68/male	Loss of weight, jaundice 1 month	Endoscopy-friable ulcerated ampulla	Endoscopic biopsy	-	-	-	-	-	Tubules	Low columnar	+++	+	Pancreatobiliary	3	+++	--	--	Pancreatobiliary	Present

12	S188/09	57/male	Jaundice, abdominal pain 1 month	Endoscopy-duodenal ulcer with a mass in the ampullary region	Whipple's specimen	Periampullary	1.5 × 1 cm	Ulcer with raised margins	Infiltration in to duodenal wall and pancreatic parenchyma	3/6	Tubules, papillae	Tall columnar	++	+	Pancreatobiliary/intestinal	2	++	1	+	Pancreatobiliary/intestinal	Present
13	S1264/07	52/male	Jaundice, loss of weight 1 month	Endoscopy-ulcer in ampullary region	Whipple's specimen	Periampullary	4 × 2 cm	Ulcer in duodenal lumen	Infiltration in to duodenal wall	3/5	Tubules, sheets, and cords	Low columnar to tall columnar	++	++	Pancreatobiliary/intestinal	3	++	1	+	Pancreatobiliary/intestinal	Present

14	S2691/08	55/male	Abdominal pain and jaundice for 2 weeks	CBD showed an area of hard stricture with occlusion of lumen	Whipple's specimen	Ampullary	1.5 cm	Edema of ampullary mucosa, stricture with occlusion of CBD lumen	Infiltration in to wall of duodenum, CBD, pancreas	1/5	Tubules	Cuboidal to low columnar	+++	++	Pancreatobiliary	1	+	--	--	Pancreatobiliary	Present

15	S3074/09	50/male	Jaundice, fever, vomiting for 3 months	Endoscopy-ampullary prominence	Whipple's specimen	Ampullary	1.5 cm	Nodular projection at ampulla	—	1/1	Tubules, papillae	Tall columnar	+	++	Intestinal	3	+++	--	--	Intestinal	x

16	S4966/09	65/male	Obstructive jaundice 4 months	CT-dilated CBD and pancreatic duct	Endoscopic biopsy	Intra-ampullary	—	Ampullary prominence	—	—	Tubules, cribriform	Low columnar	+++	++	Pancreatobiliary	1	--	--	--	Pancreatobiliary	Present

17	S2724/09	53/male	Jaundice, vomiting 15 days	CT-IHBR dilatation, mass lesion in periampullary region of CBD	Endoscopic biopsy	Periampullary	-	-	-	-	Tubules, cribriform	Tall columnar	++	--	Intestinal	1	--	1	+	Pancreatobiliary/intestinal	Present

18	S853/09	47/male	Abdominal pain, loss of weight, jaundice 2 months	USG-dilated CHD, CBD up to terminal end	Endoscopic biopsy	Periampullary	-	-	-	-	Tubules	Low columnar	+	+	Pancreatobiliary	2	--	--	--	Pancreatobiliary	Present

19	S1281/08	57/male	Obstructive jaundice 2 months	ERCP-periampullary growth	Endoscopic biopsy	Periampullary	-	-	-	-	Tubules, cribriform	Low columnar	+++	+	Pancreatobiliary	3	--	--	--	Pancreatobiliary	Present

20	S4369	66/male	Itching, loss of appetite 2 months	Endoscopy-growth in D2, dilated CBD	Whipple's specimen	Periampullary and ampullary	—	Ulceroproliferative growth	Involve adjacent duodenum pancreatic parenchyma and CBD	5/10	Papillary, cribriform	Tall columnar	+++	+	Pancreatobiliary	3	--	1	+	Pancreatobiliary/intestinal	x

21	S2193/16	60/male	Obstructive jaundice 4 months	Endoscopy ampullary growth	Endoscopic biopsy	Intra-ampullary	-	Ampullary prominence	-	-	Tubules, papillae	Low columnar	++	+	Pancreatobiliary	2	--	--	--	Pancreatobiliary	Present

22	1640/09	60/male	Itching, abdominal pain, and jaundice for 1 month	USG-mass in pancreas. Endoscopy ampullary prominence	Endoscopic biopsy	Periampullary	-	-	-	-	Tubules	Cuboidal to low columnar	++	+	Pancreatobiliary	--	-	--	--	Intestinal	x

23	558/07	52/male	Obstructive jaundice	CT-dilated main pancreatic duct and CBD up to ampulla. No mass lesion	Endoscopic biopsy	Periampullary	-	-	-	-	Tubules, papillae	Tall columnar	+	--	Intestinal	1	+	--	--	Pancreatobiliary	x
24	4267/09	70/male	Obstructive jaundice	Endoscopy-friable growth ampullary region	Endoscopic biopsy	Ampullary	-	-	-	-	Tubules, papillae	Low columnar to tall columnar	++	++	Pancreatobiliary	3	+++	--	--	Pancreatobiliary	Present

25	4084	51/male	Recurrent pancreatitis 6 months	Endoscopy ulceration in the periampullary region	Whipple's specimen	Periampullary	1 × 2 cm	Ulcerated lesion with raised borders	-	-	Tubules, cribriform, papillae	Tall columnar to cuboidal	+	+	Intestinal	1	+	2	++	Pancreatobiliary/intestinal	Present

26	4675/10	56/male	Pancreatitis, cholecystitis 1 month	Dilated IHBR	Whipple's specimen	Ampullary	1.8 × 0.8 cm	Ampullary prominence	-	2/8	Tubules	Cuboidal to low columnar	+++	++	Pancreatobiliary	3	+++	--	--	Pancreatobiliary	Present

27	3826/10	65/male	Obstructive jaundice 1 month	Moderately dilated intra- and extrahepatic biliary channels and pancreatic duct	Endoscopic biopsy	Periampullary	-	-	-	-	Tubules, cribriform	Low columnar to cuboidal (mucinous)	++	+++	Pancreatobiliary	3	+++	--	--	Pancreatobiliary	Present

28	3995/10	55/male	Obstructive jaundice	ERCP-periampullary growth	Endoscopic biopsy	Periampullary	-	Periampullary nodular growth	-	-	Tubules, papillae	Cuboidal to tall columnar	+	--	Pancreatobiliary/intestinal	3	+++	--	--	Pancreatobiliary	x

29	680/11	55/male	Obstructive jaundice	Endoscopy-periampullary growth	Whipple's specimen	Periampullary	4 × 2.5 cm	Ulceroproliferative growth	-	-	Tubules, papillae, cribriform	Tall columnar to cuboidal	++	+++	Pancreatobiliary/intestinal	3 in papillary pattern and negative in tubules	+++	2 in papillary to 3 in tubules	++ to +++	Pancreatobiliary/intestinal	Present

30	2845/11	50/male	Obstructive jaundice	Endoscopy-periampullary ulceroproliferative growth	Endoscopic biopsy	Periampullary	-	Ulceroproliferative growth	-	-	Papillae, tubules	Tall columnar	++	++	Pancreatobiliary/intestinal	--	--	3	++	Intestinal	x

**Table 3:** Specimens lacking correlation between morphological type and immunohistochemical findings.

Morphological type	Cytokeratin profile	No. of cases
Intestinal	CK7+/CK20− (pancreaticobiliary)	3
Intestinal	CK7+/CK20+ (mixed)	2
Pancreaticobiliary	CK7+/CK20+ (mixed)	1
Pancreaticobiliary	CK7+/CK20− (intestinal)	1
Pancreaticobiliary	CK7−/CK20− (undefined)	1
Mixed	CK7+/CK20− (pancreaticobiliary)	1
Total		9

**Table 4:** Number of cases by nuclear grade and cytokeratin profile.

Cytokeratin profile	Nuclear grade		
	+	++	+++
CK7+/CK20−	4	6	8
CK7−/CK20−	0	1	0
CK7+/CK20+	1	5	2
CK7−/CK20+	2	1	0
Total	20		10

**Table 5:** Number of cases by desmoplasia grade and cytokeratin profile.

Cytokeratin profile	Desmoplasia grade				
	+	++	+++	.	Total
CK7+/CK20−	7	5	2	4	18
CK7−/CK20−	1	0	0	0	1
CK7+/CK20+	3	3	1	1	8
CK7−/CK20+	1	1	0	1	3
Total	12	9	3	6	30

## Data Availability

The data used to support the findings of this study were from the laboratory archives of the Department of Pathology, PSG Institute of Medical Sciences and Research, Coimbatore, Tamil Nadu, India. The data is available from the corresponding author upon request.
